# Personality and affections in university students: implications of circadian typology

**DOI:** 10.5935/1984-0063.20220056

**Published:** 2022

**Authors:** Giovanna de Almeida Santos, Juliana Oliveira Moreira, Ana Maria Mazon Araujo, Gabriela Correia Teixeira, Nicolle Helena Carvalho Vaz, Júlia Gabriela Antunes Fonseca, Michael J. O Andrade

**Affiliations:** State University of Minas Gerais, Psychology - Divinópolis - Minas Gerais - Brazil

**Keywords:** Sleep, Personality, Students

## Abstract

The chronotype refers to the individual differences related to the preference to perform activities or to rest during the wake or in the preferences for a certain period of the day. In this study, we evaluated how the chronotype can be considered a variable of interest for individual personality differences. Still, it was verified how the positive and negative effects and self-esteem interact with the quality of sleep and the circadian personality according to the Big Five personality factors. This study included 150 volunteers of both sexes (41 men and 109 women) aged between 16 and 44 years old (M=22.08; SD=3.8 in age). The analysis of variance showed significant differences for Horne and Ostberg [F(2.148) = 401.69; η2=0.85] usual sleep efficiency [F(2.148) = 4.83; η2=0.6] and the sleep quality index [F(2.148) = 3.25; η2=13.0]. Morning subjects had better behavioral indexes of sleep quality when compared to evening subjects. Regarding positive affects [F(2.147) = 3.54; η2=0.53], morning subjects had a higher score than afternoon subjects (p=0.34) and consequently had higher scores in kindness traits [F(2,148) = 6.81; η2=0.95] and emotional stability [F(2.188) = 6.58; η2=0.91]. The chronotype is associated with personality factors and sleep behavior (efficiency and sleep latency as basic requirements for good quality of sleep) and variables such as sleep efficiency and quality of sleep can be moderators of this behavior.

## INTRODUCTION

The associations between personality traits and individual differences according to day and night preferences have been a field of investigation within the scope of chronopsychology. However, it is not clear the validity of generalization of these studies, it is still unclear whether the circadian typology affects the personality or if some third factor influences both. Chronobiology points out that circadian rhythms have two main properties, the first being the neurobiological parameters or biological clocks of circadian rhythms^1^ and, consequently, the properties of external synchronizers, too known as zeitgeber, who maintain a temporal phase relationship that contributes to the wide range of human chronotypes^2^.

Chronotypes are commonly known as circadian typology [CT]^3^, which consists of three chronotypes (morning [MT], intermediate [NT], and evening [ET])^1^. MT subjects have a preference for going to bed early and waking up early and tend to reach their peak mental and physical performance earlier in the day. In contrast, ET subjects have a preference for going to bed and waking up late, and for performing better at the end of the day and late at night. However, a large part of the adult population is classified with an intermediate chronotype^4^. There is evidence to suggest that CT is influenced by individual factors, such as age and sex^5^-^7^, environmental factors, including exposure to light^8^,^9^, and also to cognitive skills and personality traits^10^-^15^.

CT is associated with different lifestyles. Recently the effect of the five major personality factors with CT in Polish high school students^14^ was verified. They pointed out that the participants’ diurnal preference was associated with the difficulty of carrying out avoidance strategies to deal with academic stress as high school approaches the end; however, the subjects with a neurotic trait were able to deal with an aversion to classes, distracting with night activities, such as using the internet. ET subjects are associated with sleep problems, anxious/depressive symptoms, smoking, caffeine consumption, alcohol consumption, and suicidal behavior^13^. Still, it argues that the nocturnal type of both males and females have patterns inferior to the behaviors of extraversion and social desirability^16^.

Studies show that that extroversion would moderate the association between chronotype and satisfaction^11^. This hypothesis was supported for extroverted nocturnal subjects showing disproportionately high satisfaction with life, while introverted nocturnal types showed the lowest levels of satisfaction. The level of extroversion can influence whether nighttime types choose to engage in adaptive social activities that increase well-being at night.

Muro et al. (2009)^17^ used the five factors of Zuckerman (AFFM) and CT and suggested that ETs are more outgoing, impulsive, and in search of new things, while MTs tend to be more introverted, conscientious, pleasant, and emotionally stable. They also suggest that MT type subjects are more conscientious than ET subjects. ET types are more neurotic than morning ones^7^. Also, they validated their hypothesis by suggesting that women of the ET type are more impulsive and look for new actions^18^.

The relationship between CT and the Cloninger model of the seven dimensions of personality was also explored^10^. This model considers four dimensions of temperament, damage prevention, news search, reward dependence, and persistence; and three dimensions of character (self-direction, cooperativeness, and self-transcendence). The authors showed that individuals of the nocturnal type had a greater search for novelties, but lower scores for damage prevention, persistence, and self-direction. Also, CT modulated gender differences in relation to damage prevention and search for novelties, that is, only men of the nocturnal type had a lower damage prevention score.

The relationship between personality and CT shows some inconsistent results and the hypothesis has been raised that the model used to measure personality may have a moderating effect on this relationship. Also, a mediator of depression between morning behavior and the five great personality traits^19^ was investigated. The use of different tools and mediating factors has methodological limitations related to sleep quality instruments and processes^12^,^20^. Thus, this study sought to identify mediating factors such as mood and self-esteem associated with personality, quality of sleep, and circadian typology.

## MATERIAL AND METHODS

### Study place

This research was carried out by the Laboratory of Neuroscience, Behavior and Sleep Psychology (LPNeC), located at the State University of Minas Gerais. However, the data collected refer to all regions of Brazil during the period from August to September 2020, which is equivalent to the winter and spring seasons according to the geographic layout of the southern hemisphere. Photic factors and geographic location are important markers for the circadian disposition of living organisms^21^.

### Sample

This study included 150 volunteers of both sexes (41 men and 109 women) aged between 16 and 44 years old (M=22.08; SD=3.8 in age). All participants were undergraduate students, 80.1% from public institutions and 19.1% from private ones. Furthermore, 82.8% of the participants were from the southeast region of the country, 10.6% from the northeast region, and 3.4% from the southern region. Participants were subdivided into three groups according to the circadian typology: ET (n=40; M=21.8; SD=3.2); NT (n=71; M=21.73; SD=3.3); and MT (n=39; M=21.03; SD=4.9). Exclusion criteria were participants who used alcohol or illicit drugs, used psychotropics, had a known autoimmune disease that affects hypothalamic areas associated with homeostatic and circadian sleep control. In addition to neuropsychiatric mood disorders or disorders that were related to sleep or that alter the sleep-wake cycle.

### Instruments

Pittsburgh sleep quality index (PSQI): it is a subjective measure that evaluates the quality of sleep-in relation to the last month. This instrument consists of 19 items that are ordered by seven factors: 1) subjective sleep quality, 2) sleep latency, 3) sleep duration, 4) usual sleep efficiency, 5) sleep disorders, 6) use of sleeping medications, and 7) daytime dysfunction. This questionnaire has a total score of 21 points, being distributed on a scale of 0 to 3 points per item, and it demonstrates that the higher the score, the worse the quality of sleep. In this study, the translated version and the validation for the Brazilian population^22^. It should be noted that its validation in Portuguese was performed with a high degree of sensitivity (65%).

Horne and Ostberg matutinity and evening questionnaire: questionnaire prepared and validated/adapted to the Portuguese language^23^. The purpose was to assess the individual’s preference to carry out their activities during the 24 hours. The questionnaire consists of 19 questions about habitual situations in the individual’s daily life. The results classify individuals into five chronotype categories: evening (16 to 30 points); moderately evening (31 to 41 points); indifferent or intermediate (42 to 58 points); moderately morning (59 to 69 points); and morning (70 to 86 points).

Hamilton anxiety assessment scale (HAM-A): the instrument is an objective method of tracking anxiety. The version used in this study has 7 partial items and 7 general items, which in all verify mood, tension, fears, insomnia, intellectual difficulties, motor somatizations, sensory somatizations, symptoms, and behavior during the interview. They must be answered with scores from 0 to 4 (0 = absent, 1 = mild, 2 = moderate, 3 = severe and 4 = incapacitating). Its score ranges from 0 to 56, and the final result classifies individuals into 3 categories: mild anxiety (18 to 24 points); moderate anxiety (25 to 30 points); and severe anxiety (30 or more points).

Hamilton scale for depression assessment (HAM-D): the version of the instrument to be used in this study has 11 items, which verify aspects such as depressed mood, guilt, suicide, insomnia, delay, agitation, anxiety, work, and activities. The response scores increase according to the severity of the symptoms presented. In the end, the scores classify individuals in mild depression (15 to 24 points); moderate depression (25 to 30 points); severe depression (31 to 43 points); and very severe depression (44 or more points).

Inventory of the five great personality factors (IGFP-5): IGPF-5 has 44 items, structured in simple sentences that are answered in five points on a scale of Likert-type responses that vary from totally disagree (1) to agree (5). Its validation for the Brazilian population was carried out in a sample of 5,089, male and female participants aged 13 to 67 years old from five different regions of Brazil^24^. The items are grouped into five factors: openness, conscientiousness, extraversion, kindness, and neuroticism.

Positive and negative affection scale (PANAS): this instrument measures positive and negative subjective effects using 32 adjectives. Validation of factor analysis was used, which has the best solution for two orthogonal factors: positive affect (α=0.88) and negative affect (α=0.86)^23^. Their scores are evaluated on a Likert-scale format in five points, with the following gradation: “not a little”, which equals 1 point, “a little”, “more or less”, “quite” and “very much”, which equate to 5 points.

Rosenberg’s self-esteem scale (EAR): it has 11 items, which investigate global aspects of self-esteem. The version adapted of the original scale was used, which showed an internal consistency index above 0.80 for Cronbach’s alpha^25^,^26^. The response options were distributed on a four-point Likert scale: “I totally disagree”, “I disagree”, “I agree” and “I totally agree”. The higher the score obtained, the higher the respondent’s self-esteem index.

### Procedure

Initially, the contact with the participants was made out of convenience after the study was published on social networks. After agreeing with the volunteers, all instruments were adapted in the form of online platforms via forms for data collection, open access, to avoid as much contact as possible between researchers and interviewees during the COVID-19 pandemic. The division of the groups according to the circadian typology was established according to Horne & Ostberg’s Morning and Evening Identification Questionnaire. In the second moment, all the instruments used in the study were presented and explained. The participants took an average of 20 minutes to answer the scales and questionnaires and the participants were recommended to remain assiduous in their responses.

### Data analysis

Data were plotted in spreadsheets according to the CT description and categories. Descriptive and inferential statistical analyzes were performed using SPSS (Statistical Package for the Social Sciences), version 20. Descriptive analyzes were verified using frequency and measures of central trend. Data normality conditions were verified by Komogorov-Sminorv within the 95% confidence interval. Parametric analyses of variance (ANOVA One-Way) statistics were used and, consequently, multiple comparisons of factors through ANOVA. Once identified the interaction between categories through chi-square tests.

### Ethical aspects

The study was approved by the Research Ethics Committee of the University of the State of Minas Gerais (Protocol No. 38327020.8.0000.5115) complying with Resolution No. 466/12 and 510/16 of the National Health Council. Participation was voluntary, confidential and anonymous; the data obtained from the participant followed the guidelines and standards of research involving human beings.

## RESULTS

Initially, it was identified that 91.4% of the participants were under social isolation due to the COVID-19 pandemic; however, 100% of the volunteers were daily performing remote academic activities, of which 66.2% in day shift activities and 33.8% in evening shift. Still, in relation to remote education, 33.1% were students graduating in an academic course and 25.2% undergraduate students. Regarding health factors, 43% performed daily physical activities. According to the eligibility criteria, the participants were free from neuropsychiatric disorders; however, 26.5% had a first-degree relative with the presence of psychological disorders. Regarding behavioral sleep patterns, 67.5% had four of their own, of which 13.2% shared a bed with one person.

### Circadian typology and sleep quality

The analysis of variance (ANOVA) for independent groups showed significant differences for HO [F(2,148) = 401.69; η2=85.0], as expected by Horne and Osterg (1976) (Table 1). The data did not show significant differences between the CT and the participants’ sex (*p*=0.138). A cross-reference analysis was performed with Cramer V correction between the groups of circadian typologies and the periods of study of the (undergraduate, intermediate, and graduate) students and the study shift, however; teste ꭓ^2^ test showed no difference, (*p*=0.982) and (*p*=0.901), respectively.

**Table 1:** The difference in mean sleep patterns according to circadian typology.

	ET (n=40)	NT (n=71)	MT (n=39)	p
**HO**	34.44 (4.7)	50.59 (4.9)	64.44 (4.1)	0.000^*^
**C1 (subjective quality of sleep)**	1.34 (0.7)	1,47 (0.7)	1.04 (0.1)	0.514
**C2 (sleep latency)**	1.55 (0.9)	1.57 (1.0)	1.34 (0.8)	0.473
**C3 (sleep duration)**	0.63 (0.6)	0,81 (0.6)	0.70 (0.5)	0.308
**C4 (usual sleep efficiency)**	0.23 (0.4)	0.21 (0.3)	0.01 (0.0)	0.009^*^
**C5 (sleep disorders)**	1.03 (0.3)	1.13 (0.4)	1.08 (0.4)	0.825
**C6 (use of sleeping medications)**	0.50 (1.0)	0.34 (0.8)	0.21 (0.7)	0.275
**C7 (daytime dysfunction)**	1.66 (0.8)	1.55 (0.9)	1.74 (0.9)	0.542
**Total PSQI**	6.99 (3.1)	6.78 (2.7)	5.63 (1.7)	0.042^*^
**Time to sleep**	01:13 (109.4)	00:40 (105.9)	00:14 (104.9)	0.441
**Time to wake up**	09:21 (129.1)	09:20 (102.9)	08:10 (85.4)	0.186
**Latency**	30.47 (14.25)	27.53 (16.9)	20.98 (10.2)	0.013^*^
**Anxiety**	15.72(5.8)	19.30 (9.4)	18.90 (12.1)	0.143
**Depression**	9.89 (4.8)	8.99 (4.7)	7.49 (4.7)	0.080

Notes: p^*^<0.05; Pittsburgh sleep quality index PSQI; morning [MT], intermediate [NT], and evening [ET]; standard deviation in parentheses; Horne and Ostberg [HO].

ANOVA indicated a significant difference between groups for C4 [F(2,148) = 4.83; η2=0.6] and the post hoc Bonferroni test showed that MTs have better habitual sleep efficiency when compared to NTs (*p*=0.018) ETs (*p*=0.022). ANOVA also pointed out a significant difference between groups for the total PSQI score [F (2,148) = 3.25; η2=13.0], with a marginally significant difference between MT subjects when compared to ET (*p*=0.044). The data also pointed out a significant difference for sleep latency measured in minutes [F(2,137) = 4.45; η2=0.6], in which MT subjects have less latency to initiate sleep when compared to ET (*p*=0.016).

### Circadian typology, personality and affections

Table 2 presents the descriptive data related to the circadian typology and the affection and personality traits.

**Table 2:** The difference in mean self-esteem, affections, and personality traits according to the circadian typology.

	ET (n=40)	NT (n=71)	MT(n=39)	p
**Self-esteem**	21.57(2.4)	22.17(1,7)	22,02(1,4)	0.205
**Scale of affections**				
**Positive affects**	69.89 (12.9)	67.89(11.9)	63.14(9.4)	0.032^*^
**Negative affects**	44.57(10.8)	48.00(13.9)	43.05(12.1)	0.120
**Personalities**				
**Extroversion**	2.99(1.0)	3.15(0.8)	3.33(0.7)	0.199
**Kindness**	4.00(0.3)	3.87(0.5)	3.62(0.5)	0.002^*^
**Conscientiousness**	3.24(0.7)	3.32(0.7)	3.22(0.7)	0.740
**Emotional stability**	2.60(0.9)	2.69(0.8)	2.42(0.7)	0.003^*^
**Openness to experience**	3.72(0.5)	3.5(0.6)	3.67(0.5)	0.816

Notes: p^*^<0.05; morning [MT], intermediate [NT], and evening [ET]; standard deviation in parentheses.

ANOVA showed a significant difference for positive affect [F(2,147) = 3.54; η2=0.53] in which subjects with ET had a higher score than subjects with MT (*p*=0.34). Regarding CT and personality components, they observed significant differences for kindness [F(2,148) = 6.81; η2=0.95] and stability [F(2.188) = 6.58; η2=0.91]. ET (*p*=0.014) and NT (*p*=0.002) subjects had a higher score for kindness than MT subjects. Similarly, NT subjects (*p*=0.003) had a higher stability score than MT subjects.

A simultaneous analysis of joint variables was performed to verify how the usual sleep efficiency, latency and PSQI influence the mood and personality traits according to the CT. Multivariate analysis using CT as a fixed factor and controlling the PSQI showed that the positive affect (*p*=0.079) was no wlonger significant, the same did not occur with the kindness variables [F(3; 1.53) = 6.96; *p*<0.001; η2=13.0] and stability [F(3; 3.93) = 6.05; *p*=0.001; η2=11.0]. Similar to the PSQI, the sleep latency variable also showed control for positive affect (*p*=0.075), but maintained the significant difference for kindness [F(3; 1.00) = 4.26; *p*=0.003; η2=1.00] and stability [F(3; 3.30) = 4.84; *p*=0.007; η2=0.92]. Regarding the control of the habitual sleep efficiency variable, the analysis continued to point out a significant difference for positive affect [F(3; 386.1) = 2.83; *p*=0.041; η2=0.5], kindness [F(3; 1.03) = 6.96; *p*=0.007; η2=0.8] and stability [F(3; 2.84) = 6.05; *p*=0.005; η2=0.8].

Simultaneous analysis of joint variables was also carried out, controlling the students’ study period and shift. Multivariate analysis using CT as a fixed factor and controlling the study period maintained the significant difference for positive affection [F(3; 401) = 2.95; *p*=0.35; η2=0.60], the kindness [F(3; 1.08) = 4.69; *p*=0.004; η2=0.88] and emotional stability [F(3; 2.95) = 4.38; *p*=0.006; η2=0.83]. The same results occurred for positive affect [F(3; 319.6) = 2.31; *p*=0.035; η2=0.50], the kindness [F(3; 1.04) = 4.52; *p*=0.004; η2=0.86], and stability [F(3; 2.83) = 4.17; *p*=0.006; η2=0.80] when controlling the students’ study shift so much.

## DISCUSSION

Typological differences in circadian activity have been characterized as an ontogenetic factor of personality. The results of this study strengthen the hypothesis that the chronotypes of adult subjects are related to specific personality traits, for example, morning subjects are characterized by greater emotional stability and higher levels of kindness when compared to evening subjects. Another fundamental point in this comparison is that this difference is accentuated by the construction of positive effects in morning individuals. It is important to note that previous studies do not indicate the existence of an interaction of positive and negative affects with personality traits and circadian typology.

The chronotype as a human characteristic is based on self-reported daytime or nighttime preference criteria, using patterns of activity in usual waking time as indicators^27^. The influence of social zeitgebers (synchronizers) on behavior variation may be associated with clues intrinsic to the sleep-wake cycle of the circadian period in the personality structure.

The influence of sex on the distribution of morning and evening preferences among students was not observed. The differences in processes underlying the genders and the circadian timing system remain undefined^28^. That is, the discussion about circadian typology and personality traits remains contradictory. The nature of these conclusions can be assumed by future interactions, lack of consistent personality trait testing, and studies with internal reliability measures about temperament and personality traits associated with sleep behavioral patterns during the subject’s ontogeny.

The results also did not point out any associative differences between the shift and the study schedule of the participants. However, gender differences and the time arrangements for class and study shift must be taken into account in morning-evening studies that assess subjects’ behavioral or cognitive performance^28^. It is important to note that sleep delay, common among adolescents, can be attributed, at least in part, to social changes in the development of mechanisms that regulate sleep time.

It is important to consider the variety of personality definitions (Muro et al., 2009^17^), this concept depends on the theory adopted by the researcher. There is a panacea of personality theories that are used in the literature. A key point for this perspective was to use the factorial model of the “Big Five Personality Factors”, a large number of studies point to the validity and reliability of this measure in different contexts and cultures^24^,^27^,^29^. It used multiple regressions among gender, neuroticism and affability did not observe differences related to the chronotype^12^. Differently, it was pointed out that individuals with more nocturnal habits had higher scores in extraversion and kindness^29^. Similar results were found in this study; however, differences were observed for affable or loving behaviors in subjects with morning preference and, consequently, greater emotional stability for morning subjects. These results are similar to the findings^7^, which suggest that morning subjects are more conscientious than nocturnal subjects^13^. Refer that the sense of restraint and practical sense is consistent with practices of sensitivity and confidence of the kindness trait. It is important to highlight that the morning subjects had a better sleep quality index. However, we can associate these results with the hours for work and study that are socially determined, most of the time, in a daytime society. Thus, the preferences of the morning individual end up being consistent with this reality, presenting a higher quality of sleep and lower barriers in social adaptation. Thus, it is pointed out that the quality of sleep can be an important control variable to maintain a positive effect according to the CT.

Still, there is a marginal tendency for extroverted behavior for nocturnal subjects. However, this difference was not significant. The tendency towards extroversion may be related to nocturnal social synchronizers such as enthusiasm and adaptive social activities that increase well-being during the night. It pointed out that the tendency to seek stimulation would moderate the association between chronotype and satisfaction^11^,^16^.

Morning types have a higher habitual sleep efficiency than nocturnal ones, and the nocturnal ones showed longer sleep latencies. Sleep latency can also be a control variable for positive affect, that is, morning subjects with lower latencies have greater sleep efficiency. The data also point out that sleep efficiency also has a marginal tendency in associative strength with kindness and emotional stability.

## CONCLUSION

To conclude, the chronotype is associated with personality factors and sleep behavior (sleep efficiency and latency as basic requirements for good sleep quality). These constructs are mainly related to positive affect and affable or emotionally stable behaviors for morning subjects. Although this study presents new associations of CT and emotional and personality behavior, studies of this nature have contributed with ideas about social behavior around everyday social experiences, such as positive and negative affect, for example, cohabitation with family members, interaction with friends and occupational roles. Future research may focus on different methodological measures of personality measurement (e.g., temperament scales associated with behavioral neuroimaging and electrophysiological measures to check for structural sleep stages), use different chronotype measures, or objectively assess the actual sleep-wake cycle using actigraphy. Still, a three-dimensional resolution interaction between circadian typology, personality traits, and cognitive process models, for example, memory and attention models, can contribute to support the interaction of these patterns. However, it is argued that personality assessments, when treating sleep behavioral patterns and their typological variables, are of paramount socio-environmental importance.
